# An end to lifetime blood donation ban in Israel for MSM would be a major step toward a science-based policy that reduces stigma

**DOI:** 10.1186/s13584-017-0139-2

**Published:** 2017-03-23

**Authors:** Sean Cahill, Timothy Wang

**Affiliations:** 1 Health Policy Research, The Fenway Institute, Boston, USA; 2 Curriculum and Policy, National Center for Innovation in HIV Care, Boston, USA; 3 LGBT Health Policy Analyst, The Fenway Institute, Boston, USA

**Keywords:** Blood donation, HIV, MSM, Gay, Bisexual, Health policy, Individual risk assessment

## Abstract

In recent years, countries around the world have revised their blood donation policies regarding gay and bisexual men, and other men who have sex with men (MSM). The United States lifted the lifetime ban on MSM from donating blood in 2015, replacing it with a 1 year deferral policy allowing MSM to donate if they abstain from sex for 12 months. Other countries followed suit, while Italy and Spain have implemented deferral policies based on individual risk assessments regardless of sexual orientation. If Israel were to adopt a one year deferral policy for MSM, as recommended by Drs. Ginsberg et al. in this issue, the increase in risk to the blood supply would be minimal. Moving to a 1 year deferral policy would be an important step forward, but it could still be seen as stigmatizing to gay and bisexual men. We recommend that Israel consider a deferral policy based on individual risk assessment rather than a blanket deferral for all MSM. MSM can engage in low- and high-risk sexual behaviors. Those who consistently engage in low-risk behaviors, such as using condoms and pre-exposure prophylaxis consistently, pose little risk to the blood supply. An individual risk assessment policy would screen potential donors of all sexual orientations for low-, medium-, and high-risk behaviors. Potential donors identified as high-risk, such as injection drug users, would justifiably be subject to lengthy or permanent bans. MSM who engage in low-risk sexual behaviors would be allowed to donate without deferral. Medium-risk donors, such as men who have recently had unprotected anal sex with another man, would be subject to a deferral period of 1 month, which is in line with the window period of current HIV screening technology. Most fourth generation HIV tests can detect HIV within a month, and the nucleic acid test used to screen blood can detect HIV in just 9–11 days. Various studies have developed questions for ascertaining HIV risk among MSM which could be used in blood donor questionnaires. Using tablets or other technology that enhances privacy to conduct the blood donor questionnaire could improve collection of this sensitive information.

**Electronic supplementary material:**

The online version of this article (doi:10.1186/s13584-017-0139-2) contains supplementary material, which is available to authorized users.

## Background

In recent years several countries have revised their policies regarding blood donation by men who have sex with men (MSM). These changes have occurred due to advances in blood screening technology, and due to an acknowledgement of the stigmatizing nature of the lifetime MSM blood donation, and the desire of many low-risk MSM to contribute to disaster preparedness and a strengthening of the blood supply.

The United States ended its lifetime ban on MSM blood donations and adopted a 1-year deferral policy in 2015, meaning that MSM must abstain from sex for 1 year to be eligible to donate blood [1]. Canada reduced its lifetime deferral for MSM to 5 years in 2013 and to 1 year in 2016 [2]. South Africa, had a 6-months deferral policy for MSM until 2014. Now people are deferred if they have a new sexual partner in the past 6 months [3] or report multiple partners in the past 6 months [4], regardless of the sex of those partners. Russia lifted a ban on MSM donating blood in 2008, but government officials were considering reinstating the ban after passing anti-gay laws in 2013 [5]. France ended its lifetime ban on MSM blood donations in 2016. MSM who have not been sexually active with other men in the past year are now eligible to donate blood in France. Gay men who have only had one partner in the preceding 4 months, or who have not been sexually active, can donate blood plasma. The French Health Ministry will continue to conduct studies, and the deferral period for gay men will gradually be reduced if there are no increases in health risks [6].

As of 2015, Austria, Germany and Belgium still had lifetime bans for MSM who wish to donate blood [6]. The lifetime ban on MSM donating blood was lifted in 2011 in England, Scotland, and Wales, and in 2016 in Northern Ireland. It was replaced with a 1-year deferral period for sexually active MSM. The British government is now conducting a review to see if the deferral policy should be shortened [7]. Japan, the Netherlands, Australia and New Zealand all have 1-year deferrals for MSM blood donation [6]. In Italy and Spain, donors are screened for high-risk sexual behavior regardless of the sex of their partners or their sexual orientation. Deferrals are made based on individual risk [6].

## Commentary

In their paper in the current issue of the *Israel Journal of Health Policy Research* [8], Drs. Ginsberg, Shinar, Kopel and Chemtob analyze this important public health policy, which currently bans Israeli men from donating if they have had sex with another man since 1977, and recommend a shift to a 1-year deferral. In other words, an MSM who seeks to donate would have to abstain from sexual activity for 1 full year before donating. Such a change in policy would result in a minimal increase in risk in Israel—1 transfusion transmission incident, or TTI, per century. The increased risk estimate from moving from a lifetime MSM ban to a 5-years deferral policy in Israel would be 1 TTI over 2 centuries. However, Ginsberg et al. estimate that a complete end to the MSM blood donation ban with no deferral period during which an MSM donor had to abstain from sex would lead to a six-fold increase of risk of a TTI: 4.99 TTIs over the next decade.

Were the Israeli government to implement the Ginsberg et al.’s recommendation, this would represent an important incremental step towards a science-based policy which maintains the safety of the blood supply without stigmatizing gay and bisexual men. While no one has a right to donate blood, and therefore the current policy is not discriminatory in the way anti-gay discrimination in employment or housing is, a change in policy to allow some low-risk MSM to donate blood would reduce stigma against MSM, and be in line with Israel’s relatively enlightened treatment of sexual minorities under public policy, especially in contrast how gay, lesbian and bisexual people fare in other countries in the Middle East [9].

The nucleic acid test (NAT) used to screen blood can detect HIV in just 9–11 days after infection [10]. New technological advances greatly decrease the risk of HIV-infected blood escaping detection; however, they cannot completely eliminate the risk of HIV in the blood supply. Therefore, NAT technology should be used in conjunction with comprehensive individual risk assessments that can adequately screen potential donors for low- and high-risk sexual behaviors.

A lifetime ban on blood donations by MSM, which is Israel’s current policy and which was the policy of the United States until late 2015, is based on a flawed understanding of male same-sex behavior. Sexually active gay and bisexual men who are at low risk (i.e. who are monogamous with an HIV-negative partner, who use condoms and lubricant, or who don’t have receptive anal intercourse without a condom) are not allowed to donate. Many gay men have sex but don’t have condomless anal sex. Most gay and bisexual men are HIV-negative [11], and most are not at high risk of HIV infection, yet they are denied the ability to donate blood under the current lifetime ban. A meta-analysis by Beyrer, Baral, van Griensven et al. found that HIV prevalence among MSM ranges from a low of 3.0% in the Middle East and North Africa region to 25.4% in the Caribbean. Prevalence among MSM in Western Europe is 6.1%, in Eastern Europe and Central Asia 6.6%, and in North America 15.4% [11].

Israel should consider going beyond the recommendations of Ginsberg et al., and consider a deferral policy based on individual risk assessment rather than a blanket deferral for all sexually active MSM. A more rational policy based on individual risk assessment would identify low-, medium-, and high-risk potential donors. Low-risk MSM, such as those who have not had any anal sex recently or those who exclusively used condoms during sex, would be allowed to donate without deferral. High-risk potential donors of any sexual orientation, such as those who ever injected drugs or performed commercial sex work, would continue to be subject to the lifetime ban on donating blood as indicated by the current United States protocol [1]. Potential MSM donors who are identified as medium-risk, including those who have engaged in higher risk sexual behaviors such as recent unprotected anal sex, would be subject to a 30-days temporary deferral before being allowed to donate.

Often donor history questionnaires do not adequately distinguish between lower and higher risk sexual behaviors by MSM donors or others. Both MSM and non-MSM donors can engage in low-risk sexual behaviors, or high-risk sexual behaviors. In addition, certain sexual acts are more high-risk for acquiring HIV than others (see Additional file 1: Table S1) [12]. For example, receptive anal intercourse without protection from condoms and lubricant and/or pre-exposure prophylaxis (PrEP) is much higher risk than oral intercourse.

The most effective questions for identifying individuals at risk of transmitting HIV through blood donation would screen out potential donors who engage in high-risk sexual behaviors. Questions to identify the risk of potential donors already exist in the U.S. donor history questionnaire. To differentiate between low- and medium-risk MSM donors, the individual risk assessment questions should focus on recent (within 2–4 weeks) sexual history. Low risk donors would include, for example, those who have not had any recent anal sex and those who consistently use condoms and/or PrEP [13]. Low risk MSM should be allowed to donate without a temporary deferral. MSM donors that are determined to be medium risk should be subject to a short deferral period. Based on epidemiological research and U.S. Centers for Disease Control and Prevention (CDC) recommendations, criteria for being classified as medium risk can include:

- having multiple, casual male partners in the last 2–4 weeks
- having any unprotected anal sex with a man in the last 2–4 weeks
- having 1 or more HIV-positive partners in the last 2–4 weeks
- having a recent diagnosis or history of gonorrhea, chlamydia, and/or syphilis

MSM donors determined to be medium risk could be subject to a temporary deferral period of 30 days. Deferral periods that are substantially in excess of known window periods provide little additional value to ensuring disease detection [14]. Different studies have estimated the window period for various fourth-generation HIV tests to be approximately 2 weeks to 1 month in length [15]. Therefore, after a deferral period of 30 days, potential donors who are HIV-positive should be detected by current HIV testing technology.

The U.S. CDC and the U.S. Public Health Service released PrEP guidance in 2014 [16]. In a supplement for providers, a risk index tool is provided “to quickly and systematically determine which MSM are at especially high risk of acquiring HIV infection” [16]. This risk index contains several questions for determining high-risk of acquiring HIV (see Additional file 1: Table S2) [16].

The MSM Risk Index was based on several epidemiological studies. One study developed and validated a prediction model for HIV acquisition among MSM based on medical records data from a United States sexually transmitted diseases (STD) clinic from 2001 to 2008. The predictive model generates a risk score based on previous history of STDs, drug use, sex with HIV-positive partners, and number of sexual partners. The study provided a simplified risk score estimation tool that includes specific questions for ascertaining high HIV risk (see Additional file 1: Table S3) [17].

The questions that are recommended by the CDC and the U.S. Public Health Service in their PrEP guidelines ask about specific high-risk sexual practices. These questions were designed specifically for MSM, so they should be understandable and acceptable to potential MSM donors. Blood donation centers should ask all potential donors about high-risk behaviors, but they could also structure their questionnaire such that men who indicate that they have sex with other men are asked a particular set of questions such as those described above.

The blood bank industry should consider administering donor risk questionnaires using tablets, such as iPads, which convey a greater sense of confidentiality and could lead to more accurate reporting of risk data and a greater ability to screen out high-risk would-be donors [18]. Reassuring all donors that any information provided on the donor history questionnaire will be kept confidential, and using technologies that enhance a sense of privacy, can facilitate the collection of sensitive data. Research has shown that use of technologies that minimize responding directly to a questioner has been shown to facilitate the collection of sensitive data, including sexual orientation, substance use, and mental health data. Respondents to a sexual health survey who used telephone audio computer-assisted self-interviewing (T-ACASI) instead of human interviewers were 1.5–1.6 times more likely to report same-gender sexual attraction, experience, and genital contact. The impact of T-ACASI was more pronounced (odds ratio = 2.5) for residents of communities that were less accepting of homosexuality and for respondents who were parents raising children (odds ratio = 3.0) [19]. A related technology is the use of electronic patient-reported outcomes (ePRO) tablets in clinical settings. ePRO tablets have been shown effective in collecting sensitive information from HIV patients, including injection drug use, depression, and treatment adherence data [18]. Given the experience with T-ACASI and ePRO, it is likely that the use of tablet technology to administer the donor history questionnaire would lead to more accurate responses to individual risk assessments, thereby increasing the ability of blood banks and other blood donation centers to screen out potential high-risk blood donors.

Because these individual risk assessment questions are sensitive in nature, it will be necessary to train staff who will be working with potential donors in cultural competency to do a sexual history with a gay or bisexual man. The Fenway Institute at Fenway Health in Boston, U.S. [20], and the (U.S.) National LGBT Health Education Center offer resources and training on LGBT cultural competency [21].

## Conclusions

An end to Israel’s lifetime blood donation ban for MSM would be a major step toward a science-based policy that reduces stigma for gay and bisexual men. Effective risk behavior questions exist that could allow for an individual risk assessment to allow low-risk MSM to donate with no deferral.

## Additional file

Additional file 1: Table S1. Estimated per-act risk for acquiring HIV from an infected source, by exposure act, CDC. **Table S2.** HIRI-MSM Risk Index. Smith et al., *JAIDS*, 2012. **Table S3.** Simple Risk Score Estimation, Menza et al., *Sex Trans Dis*, 2009. (DOC 350 kb)

## Abbreviations

ePRO: electronic patient-reported outcomes
HIV: Human immunodeficiency virus
LGBT: Lesbian, gay, bisexual, transgender
MSM: Men who have sex with men
NAT: Nucleic acid test
PrEP: Pre-exposure prophylaxis
STD: Sexually transmitted disease
T-ACASI: Telephone audio computer-assisted interviewing
