# Supplementary material for: An end to lifetime blood donation ban in Israel for MSM would be a major step toward a science-based policy that reduces stigma
Source: Isr J Health Policy Res. 2017 Mar 23;6:15. doi: 10.1186/s13584-017-0139-2 (PMC5363038; doi:10.1186/s13584-017-0139-2)
Supplement: Additional file 1: Table S1. — Estimated per-act risk for acquiring HIV from an infected source, by exposure act, CDC. Table S2. HIRI-MSM Risk Index. Smith et al., JAIDS, 2012. Table S3. Simple Risk Score Estimation, Menza et al., Sex Trans Dis, 2009. (DOC 350 kb) [file 13584_2017_139_MOESM1_ESM.doc]

**Additional file 1**

**Table S1.** Estimated per-act risk for acquiring HIV from an infected source, by exposure act, CDC.[[1]](#endnote-2)


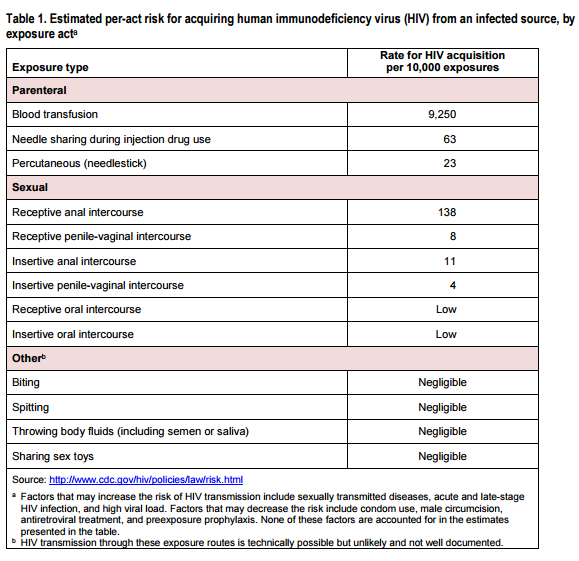


Table S2. HIRI-MSM Risk Index. Smith et al., *JAIDS, 2012.*^ii^


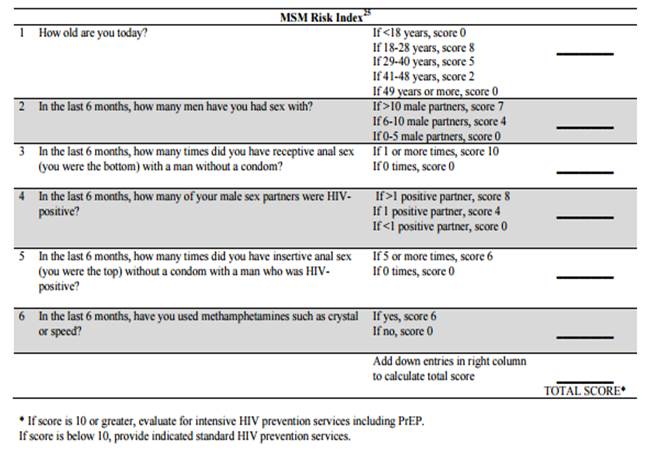


Table S3. Simple Risk Score Eatimation Menza et al., Sex Trans Dis, 2009.^iii^


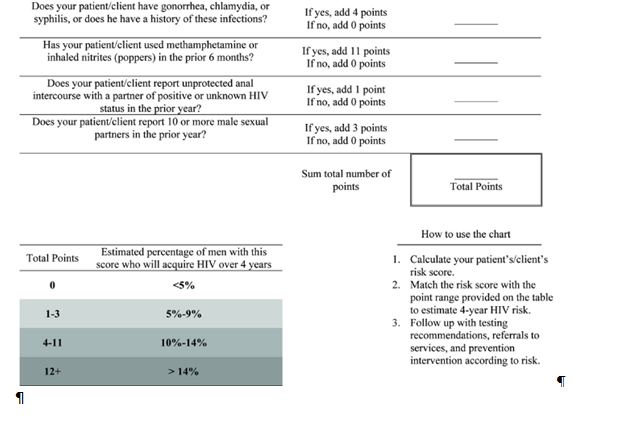


1. This graphic assumes that condoms, lubricant, and pre-exposure prophylaxis were not used.

   ^ii^ Smith D et al. 2012. “Development of a clinical screening Index Predictive of Incident HIV Infection Among Men Who Have Sex With Men in the United States.” *JAIDS*. August 2012. 60(4): 421-427.

   ^iii^ *Ibid.* [↑](#endnote-ref-2)
